# Rapid and Visible Efficacy of a Dermocosmetic in Acne Patients With Fair Skin Phototypes: Results of a Randomized Split‐Face Study

**DOI:** 10.1111/jocd.70641

**Published:** 2026-01-05

**Authors:** Catherine Queille‐Roussel, James Odeimi, Margot Broallier, Delphine Kerob, Jerry Tan

**Affiliations:** ^1^ CPCAD, CHU l'Archet Nice France; ^2^ La Roche‐Posay Laboratoire Dermatologique France; ^3^ Department of Medicine and Windsor Clinical Research Inc Western University Windsor Canada

**Keywords:** acne, dermocosmetic, fair skin phototypes, kinetics

## Abstract

**Introduction and Objectives:**

Dermocosmetics have proven their benefit in acne management. However, only very few studies investigated their efficacy kinetics. This study assessed the efficacy kinetics of a dermocosmetic cream (DC) containing *Punica granatum* Pericarp extract, Salicylic acid, Niacinamide, Zinc gluconate, and *Aqua Posae Filiformis* in subjects with acne.

**Material and Methods:**

16 subjects (14 women, 2 men; mean age 29.4 ± 7.7 years; phototype II and III) with mild to moderate acne participated in a randomized, intra‐individual, split‐face study for 15 days. Hemi‐faces randomly received DC or remained untreated. Assessments included lesion counts, acne severity, and local tolerance; subjects also rated the perceived benefit of the DC. Efficacy was also evaluated using standardized multi‐modality full‐face imaging and a mobile connected imaging system.

**Results:**

The inflammatory lesion count significantly (*p* ≤ 0.05) decreased with DC at Day 8 and Day 15; a significant decrease of the non‐inflammatory lesion count was observed at Day 15 (*p* < 0.01). The total lesion count had significantly (*p* ≤ 0.05) decreased with DC at Day 8 and Day 15. Between‐side differences were significant (all *p* ≤ 0.05) for all lesion types at D15. Local tolerance was good. Most subjects considered that their skin appearance and the visibility of acne lesions had improved.

**Conclusion:**

This exploratory study provides strong evidence on the efficacy kinetics of a DC cream in acne management in subjects with fair skin tones. It shows that early, daily and specific treatment with a targeted DC significantly improves all acne lesions type, as well as acne severity as soon as 15 days.

## 1 Introduction

*Acne vulgaris* is one of the most common chronic inflammatory skin diseases worldwide. While observed across all skin phototypes and predominantly affecting adolescents with nearly 85% experiencing it, acne is increasingly prevalent among adults [1].

If not adequately treated, acne may cause sequelae such as post‐inflammatory erythema, especially in fair skin phototypes, as well as hyperpigmentation, and scars, that can heavily impact the patient's quality of life [2].

Even though much data about the development of acne from comedones to papules, pustules, and scars is available from the literature, there is only a paucity of information available about acne lesion lifecycle when being treated or remaining untreated [3].

A key objective in acne treatment is to enhance patient adherence, which can be improved by simplifying treatment regimens to manage existing lesions, prevent permanent marks, and minimize recurrence. While current topical pharmacological acne treatments are effective, their onset of action varies, and they are frequently associated with side effects such as desquamation, irritation, or erythema [4, 5, 6]. Therefore, the development of topical products that offer a rapid onset of action, coupled with excellent local tolerance, thereby optimizing patient adherence and outcomes, is crucial. Topical treatments and dermocosmetics are widely recommended in patients with mild to moderate acne [7, 8, 9].

This study, conducted over 15 days, assessed the kinetics of acne lesions progression in response to a dermocosmetic cream (Effaclar Duo+, La Roche‐Posay Laboratoire Dermatologique, France, hereafter DC) containing multi‐targeted ingredients— *Punica granatum* Pericarp extract, Salicylic acid, Niacinamide, Zinc gluconate, and *Aqua Posae Filiformis*, compared to no treatment in adults with fair skin tones and mild to moderate acne. All ingredients have shown their benefit as monotherapy in improving acne lesions as well as acne‐induced hyperpigmentation (AIH) [10, 11, 12, 13, 14].

## 2 Material and Methods

This non‐interventional, exploratory, randomized, intra‐individual study did not require approval from an ethics committee prior to its start. Nevertheless, the study conformed to all local legal requirements for the conduct of this type of study and complied with the Principles of the Declaration of Helsinki. Moreover, all subjects who participated in this study provided written informed consent prior to inclusion.

Adult subjects with a fair skin phototype defined as phototype I, II, or III, and mild to moderate acne on both hemi‐faces according to the GEA grading system were recruited [15]. Hemi‐faces of suitable subjects were randomized to either DC (Effaclar Duo+, La Roche‐Posay Laboratoire Dermatologique, France) twice daily at home or remained untreated for 15 days. The entire face was to be cleansed with a neutral cleanser (Toleriane Purifying Cleanser, La Roche‐Posay Laboratoire Dermatologique, France) once daily at home in the morning prior to product application.

Clinical assessments at baseline, each day starting at baseline (D1) to Day 6, as well as on Day 8, 11, and 15, included inflammatory, non‐inflammatory, and total lesion counts on both hemi‐faces, acne severity using GEA, and local tolerance to the DC [15, 16]. Moreover, desquamation, dryness, and erythema, as well as burning and itching sensations related to DC use, were assessed at Day 15. In addition, subjects rated the perceived benefit of the DC using a self‐assessment questionnaire at Day 15. Images using Colorface (Newtone, France) were taken at baseline until Day 6, and at Days 8, 10, 12, and 15. Images with SkinCam (Newtone, France) were taken at baseline, Days 2–6, Days 8–13, and Day 15.

Quantitative parameters were analyzed using a mixed‐effect model. This model included: Time point, Treatment, the interaction Time point *Treatment, and baseline as fixed effects, and subject added as a random effect. The comparisons between post‐baseline time‐point and baseline for each treatment were performed using a Dunnett adjustment. The change from baseline was also analyzed using a mixed‐effect model. The comparison between treatments at each time‐point was performed regardless of the interaction results using a Tukey adjustment. The analysis of the questionnaire was descriptive only. The probability level was set at 5%.

All statistical analyses were performed using R Software version 4.0.2.

## 3 Results

### 3.1 Demographic and Baseline Data

Sixteen adult subjects (14 women, 2 men; mean age 29.4 ± 7.7 years) with phototype II (5; 31.2%) or III (11; 68.8%), and mild (11; 68.8%) or moderate (5; 31.2%) acne were included in the study.

At baseline, mean inflammatory lesion counts on the to‐be‐treated side were 14.6 ± 6.7 and 14.3 ± 7.4 on the untreated side; the mean non‐inflammatory lesion counts were respectively 12.5 ± 8.9 and 11.9 ± 7.8, and the mean total lesion counts were 27.1 ± 11.2 and 26.3 ± 9.7.

### 3.2 Clinical Efficacy

The inflammatory lesion count significantly decreased with DC at Day 8 (11.2 ± 6.0; −23.1%; *p* < 0.05) and Day 15 (9.2 ± 5.5; −36.9%; *p* < 0.001); a significant decrease of the non‐inflammatory lesion count was also observed at Day 15 (4.8 ± 9.3; −29.0%; *p* < 0.01). The total lesion count had significantly decreased with the DC at Day 8 (21.7 ± 11.2; −19.8%; *p* < 0.01) and at Day 15 (18.1 ± 10.6; −33.3%; *p* < 0.001). No significant changes in the different lesion counts were observed on the untreated side compared to baseline.

**FIGURE 1:**
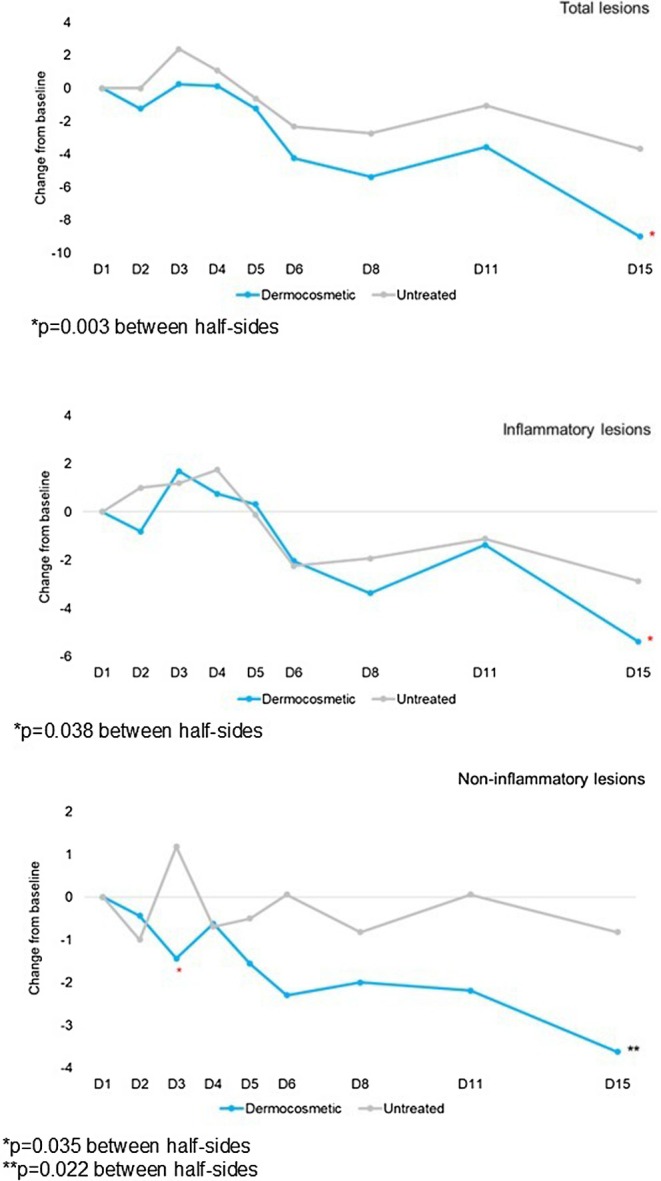
Change from baseline in the total, inflammatory, and non‐inflammatory lesion count.

A significant (*p* = 0.038) between‐side difference for inflammatory lesions, in favor of the DC, was observed at Day 15, and a significant between‐side difference for non‐inflammatory lesions in favor of the DC was observed at Day 3 (*p* = 0.035) and Day 15 (*p* = 0.022). A significant (*p* = 0.003) between‐side difference for the total lesion count in favor of the DC was observed at Day 15 (Figure 1).

Acne severity had almost cleared in 2 subjects at Day 11 and Day 15.

The investigator did not observe any desquamation, dryness, or erythema in 93.8%, 81.2%, and 87.5% of the subjects, respectively.

81.2% of the subjects reported not experiencing any burning or itching sensations after Day 15 of DC use.

**FIGURE 2:**
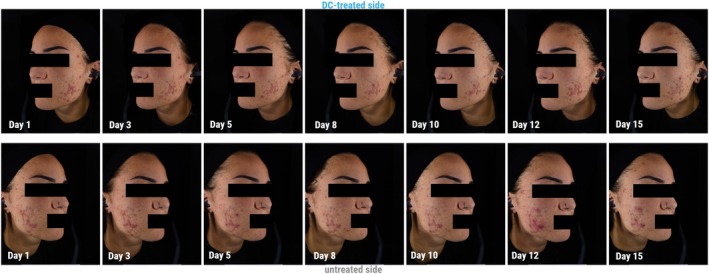
Treatment kinetics over time (Phototype III) using Colorface (best case).

**FIGURE 3:**
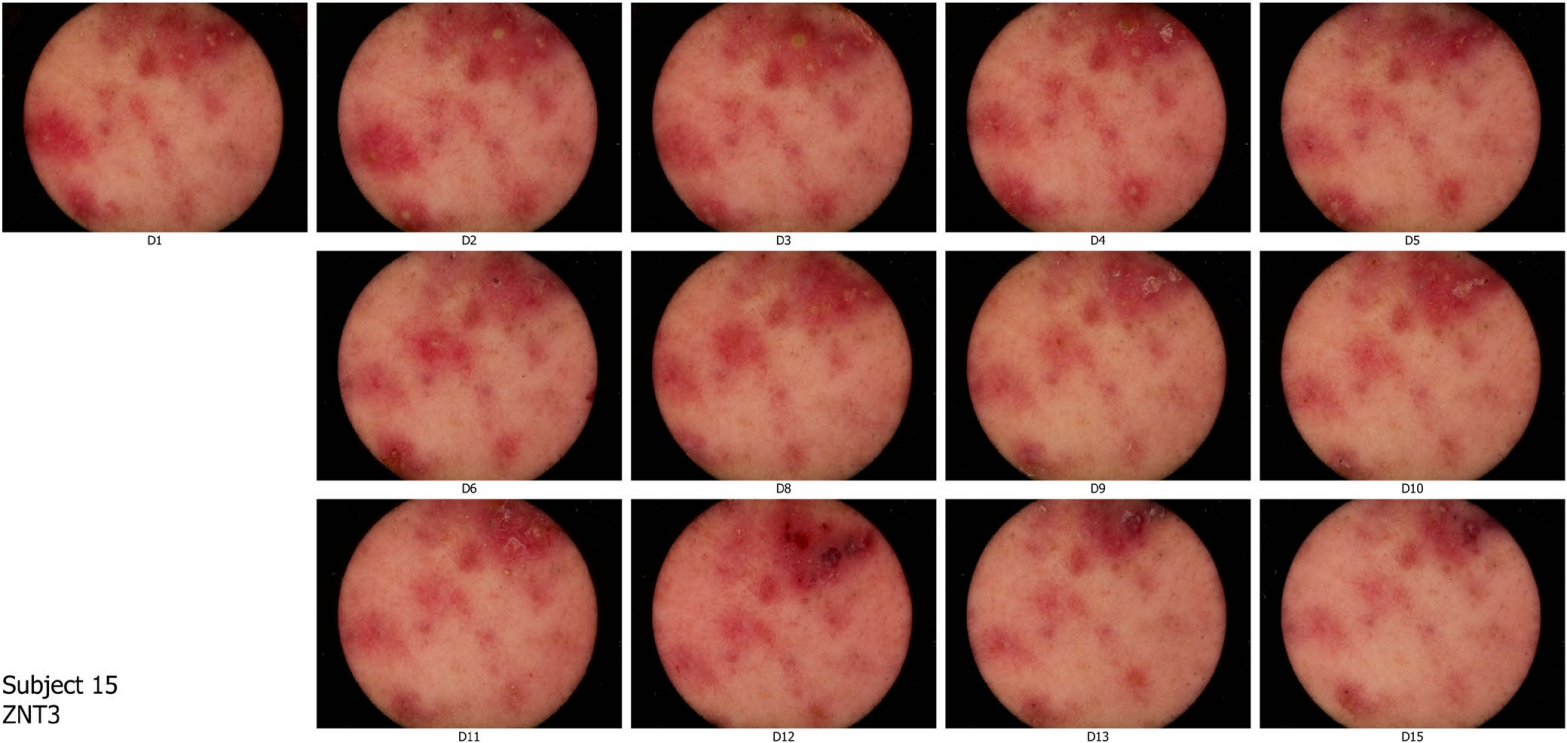
Treatment kinetics over time of DC‐treated side (Phototype III) using SkinCam (best case).

Figure 2 provides an example of the evolution over time of acne lesions kinetics in a subject using Colorface, and Figure 3 provides an example of the progression of acne lesions kinetics over time with DC treatment, using SkinCam.

94.0% of the subjects claimed that their skin appearance had improved, and that the DC is suitable for their skin type while being comfortable; 88.0% reported that the visibility of their acne lesions had improved after 15 days.

## 4 Discussion

This exploratory, intra‐individual, randomized study assessed the efficacy kinetics of a specifically developed DC containing a triple combination of salicylic acid, niacinamide, and zinc in subjects with mild to moderate acne presenting Phototype II and III over a period of 15 days, which may be considered an acute flare‐up treatment period for rapid acne lesions improvement.

Currently, clinical trials usually assess the efficacy of medications and dermocosmetics on the lesion evolution on a weekly basis and up to 12 weeks or 84 days. But until today, no study has assessed the efficacy kinetics of treatment on a daily basis during the first 15 days. During these first 15 days, management of active acne lesions may allow reducing the risk of the development of microcomedones, which are precursors of acne lesions as well as acne flare‐ups that may evolve into more severe, inflammatory forms of acne, becoming more difficult to manage and requiring pharmacological active topical or systemic treatments [17]. Thus, a product that may act as an “SOS” care, helping to rapidly reduce the risk of the development of microcomedones, acne flare‐ups, and even to reduce the number of active acne lesions without bearing the risk of local product‐related side effects such as erythema and skin dryness, may be considered an ideal immediate and intensive solution.

The present study showed that a multi‐targeted, unique dermocosmetic combining salicylic acid, niacinamide, and zinc was able to significantly (*p* < 0.001) reduce inflammatory and total acne lesion counts as early as 8 days of application and initiate the lesion healing process during the first 15 days of treatment, confirming that specific acne dermocosmetics have their place in the early management of acne [18]. Surprisingly, while all 3 ingredients are well known for their benefits in acne, currently, dermocosmetic formulations that are indicated in the treatment of mild to moderate acne only combine 2 of these 3 actives, and only scientific data for combinations of salicylic acid and niacinamide are available [19].

For decades, pharmacological treatments, especially retinoids and topical antibacterials, were the gold standard therapy for mild to moderate acne. More recently, this paradigm was nuanced to include the role of dermocosmetics in providing rapid and visible beneficial results in milder forms of acne, with less potential for local treatment‐related side effects such as dryness, erythema, burning, and desquamation [14, 20]. In 2025, an international consensus recommendation suggested that, in monotherapy, dermocosmetics developed to specifically manage acne may be beneficial in the treatment of mild to moderate forms [9]. Based on these recommendations and on our study results, we consider that the tested dermocosmetic provides substantial immediate benefits to individuals with mild‐to‐moderate acne.

Despite these positive results, this exploratory study has some limitations, including a small sample size that may not allow for generalizing the obtained study results to a larger population. However, the intra‐individual design with the controlled application of the product at the investigative site ensured proper application and compliance, and a clinical study with a larger sample size may be helpful to confirm the observed results.

In conclusion, this exploratory split‐face study provides a first glance at the acne lesion kinetics and shows that early, daily and targeted acne management significantly and rapidly improves inflammatory and non‐inflammatory acne lesions visibility as well as acne severity in subjects with fair skin tones as soon as 15 days.

## Author Contributions

M.B., J.O. and D.K. designed the study and supervised its conduct. C.Q.‐R. conducted the study. J.O. wrote the manuscript and coordinated the different writting activities with the authors and the journal. All authors analysed the data, provided input, read and approved the manuscript.

## Funding

This study was sponsored by La Roche‐Posay Laboratoire Dermatologique, France.

## Ethics Statement

This non‐interventional study did not require approval from an ethics committee prior to its start. Nevertheless, the study conformed to all local legal requirements for the conduct of this type of study and complied with the Principles of the Declaration of Helsinki. Moreover, all subjects who participated in this study provided written informed consent prior to inclusion.

## Conflicts of Interest

All authors, apart from C.Q.‐R. and J.T., are employees of La Roche‐Posay Laboratoire Dermatologique. C.Q.‐R. has no conflicts of interest to disclose. J.T. is a consultant and speaker for La Roche Posay Laboratoire Dermatologique, France.

## Data Availability

The data that support the findings of this study are available from the corresponding author upon reasonable request.
